# Socio-demographic determinants of myelofibrosis outcomes in an underserved center and the SEER national database

**DOI:** 10.1007/s00277-024-05894-7

**Published:** 2024-07-24

**Authors:** John Yan, M. Bakri Hammami, John X. Wei, Nishi Shah, Mendel Goldfinger, Ioannis Mantzaris, Noah Kornblum, Kira Gritsman, Alejandro Sica, Dennis Cooper, Eric Feldman, Marina Konopleva, Kith Pradhan, Rahul Thakur, Charan Vegivinti, Asma Qasim, Amit Verma, Swati Goel

**Affiliations:** 1 Department of Internal Medicine, Montefiore Medical Center, Bronx, NY USA; 2 Department of Medicine, Jacobi Medical Center, Bronx, NY USA; 3 Department of Hematology/Oncology, Montefiore Medical Center, Bronx, NY USA; 4 Department of Medicine, Cleveland Clinic Abu Dhabi, Abu Dhabi, UAE; 5 Department of Oncology, Montefiore Einstein Comprehensive Cancer Center, 3411 Wayne Avenue, Bronx, NY 10467-2509 USA

**Keywords:** Myelofibrosis, Social determinants of health, Myeloproliferative neoplasms, Epidemiology, Ethnicity

## Abstract

The influence of demographic characteristics and social determinants on cancer outcomes is widely recognized in various malignancies but remains understudied in myelofibrosis (MF). This study aims to investigate social and demographic variables associated with MF survival. We retrospectively reviewed data of biopsy-proven MF patients from the Surveillance, Epidemiology and End Results (SEER) database (2000–2021) and Montefiore Medical Center (2000–2023), an underserved inner-city hospital. The SEER cohort included 5,403 MF patients and was predominantly Non-Hispanic (NH) White (82%) with a median age of 69 years. The age-adjusted incidence rate of MF was 0.32 cases per 100,000 person-years, increasing annually by 1.3% from 2000 to 2021. Two- and five- year overall survival rates were 69% and 42%, respectively. Worse cause-specific survival was associated with older age, male sex, and diagnosis before 2011 (year of Ruxolitinib approval). NH-Black ethnicity, unmarried status and lower median income were independent predictors of worse overall survival. The single-center analysis included 84 cases, with a median age of 66 years. NH-White patients comprised 37% of the sample, followed by NH-Black (28.5%). Two- and five- year overall survival rates were 90% and 61%, respectively, with NH-Black patients exhibiting the lowest median survival, although the difference was not statistically significant. Age was a significant predictor of worse survival in this cohort. NH-Black and Hispanic patients lived in areas with higher socioeconomic and demographic stress compared to NH-White patients. Overall, this study highlights the association of social and demographic factors with MF survival and emphasizes the need for equitable healthcare and further exploration of social-demographic factors affecting MF survival.

## Introduction

It is well recognized that demographic characteristics and social determinants of health (SDH) such as healthcare accessibility, economic stability, and environment have significant impacts on cancer outcomes [1, 2]. Research in malignancies like acute myeloid leukemia, colorectal cancer, lung cancer, prostate cancer, and others suggests that the influence of demographic and SDH extends throughout the entire timeline of disease, from pathogenesis to treatment and survival [3–6]. However, the impact of SDH has not been as thoroughly explored in myelofibrosis (MF), a class of BCR-ABL1 negative myeloproliferative neoplasm (MPN) characterized by hematopoietic defects and risk for progression to leukemia [7]. A multi-center comparative analysis between Caucasian and African American (AA) patients found inferior post-transplant outcomes in the AA cohort [8]. Another retrospective study (*n* = 300) of MPN found inferior survival in their non-Caucasian cohort [9]. Regarding gender disparities, Barraco et al. found that females have lower risk of progression to secondary MF, lower rates of complex karyotypes, and improved survival compared to men [10]. To date, there are few population-based studies of MF which investigate demographic and social variables associated with survival, and even fewer which are representative of racially diverse cohorts. This study aims to (1) investigate socio-demographic variables associated with survival of MF patients in the Surveillance, Epidemiology and End Results (SEER) national database and a unique inner-city multi-ethnic cohort, and (2) to investigate demographic and clinical factors in the single-center cohort which vary between ethnicities.

## Methods

This study collected and analyzed data from the SEER database and from Montefiore Medical Center (MMC). The SEER program is sponsored by the National Cancer Institute (NCI) and collects comprehensive data on cancer incidence and mortality from population-based cancer registries, covering approximately 48% of the United States population (http://seer.cancer.gov/seerstat). Data for this study were obtained from the SEER-17 registries (November 2022 submission) using SEER*Stat software (version 8.4.3). The data acquisition date was August 15th, 2023. Data were examined from 2000 to 2021 to identify patients diagnosed with MF. We included patients with the International Classification of Disease for Oncology (3rd edition) codes 9961. We excluded cases lacking histological confirmation or active follow-up. We also excluded patients with death certificate or autopsy reports only.

Race was categorized as Non-Hispanic (NH) White, NH-Black, NH-Asian/Pacific Islander, NH-Alaska Native/American Indian, NH-Others, or Hispanic of all races. Year of diagnosis was categorized into those diagnosed before 2011 and those diagnosed in or after 2011, the year of approval of Ruxolitinib for treatment of MF. Other variables analyzed included sex, age at diagnosis, staging, follow-up, and median income as a surrogate for socioeconomic status. We also obtained marital status, receipt of chemotherapy and radiotherapy, cause of death and SEER registry. The type of chemotherapy received is not available on SEER database. Other important variables unavailable on SEER include genetic mutations, Dynamic International Prgonstic Scoring System (DIPSS) score, and lab values at diagnosis or on follow up. The outcome variable was categorized as “Alive”, “Dead attributable to this cancer diagnosis” or “Dead from other cause”. For the cause-specific survival analysis, patients were censored if they were alive at the end of the study period or if they died from causes other than those attributed directly to PMF. All causes of death were included for the overall survival analysis, and patients were censored only if they were alive at the end of the study period. These censoring criteria ensured that the analyses appropriately reflected the distinct endpoints being studied in the context of the sociodemographic variables analyzed, allowing for accurate interpretation of both cause-specific and overall survival outcomes.

For the single center cohort, we manually retrospectively reviewed the charts of 88 biopsy-proven MF patients referred to MMC between 2000 and 2023. Demographic, clinical and genetic/molecular data were collected, and complete case analysis was performed. 4 cases were excluded due to non-reported ethnicity. 74 patients had next generation sequencing molecular data available. Race was categorized as Non-Hispanic (NH) White, NH-Black, NH-Asian/Pacific Islander, and Hispanics of all races. No NH-Alaska Native/American Indians were observed within the single center cohort. DIPSS scores were calculated from time of diagnosis or referral. Information regarding the social vulnerability index (SVI) for each patient’s respective census tract was also collected via inputting patient addresses into the publicly available Centers for Disease Control and Agency for Toxic Substances and Disease Registry (CDC/ATSDR) interactive database tool (https://www.atsdr.cdc.gov/placeandhealth/svi/interactive_map.html) [11]. The SVI is a composite measure that ranks each census tract on 15 social factors, including poverty, lack of vehicle access, and crowded housing, which are grouped into four themes: socioeconomic status, household composition and disability, minority status and language, and housing type and transportation. The index ranges from 0 to 1, with higher scores indicating greater vulnerability. Regarding the single-center survival analysis, outcome variables were categorized as “dead” or “alive/lost to follow up,” with the latter being censored for survival analyses. Deaths were considered as events for regression analysis.

### Statistical analysis

Data analysis was conducted via R-Studio (v4.3.2) and IBM SPSS statistical package v.27 (SPSS Inc., Chicago, IL). Descriptive statistics including mean, standard deviation and percentages were used to outline patients’ characteristics. Overall survival (OS) was calculated from the time of diagnosis to the time of event or last follow-up. The effects of categorical variables on survival were assessed using Kaplan-Meier Product limit curves and using the log-rank test. The effects of continuous and categorical variables on survival were assessed using Cox proportional hazards regression. After univariate analysis was performed, factors with p value < 0.05 were entered into a multivariate model. Proportional hazards assumption was evaluated. Hazard ratio was reported for all significant factors in the model. Chi-square test was used to compare categorical variables, whereas Fischer’s exact test was used when the frequency was less than 5. T-test and ANOVA tests were used as appropriate to compare continuous data. Kruskal-Willis test was used instead of ANOVA when data were not normally distributed. All statistical tests were two-sided. A p-value less than 0.05 was considered statistically significant. SEER*Stat software (version 8.4.3) was used to calculate the incidence rates and the annual percentage change (APC). t-test was used to determine if the APCs were statistically significant. Rates were adjusted to the 2000 US standard population and expressed by 100,000 person-years.

## Results

### SEER cohort

The SEER analysis included 5403 patients diagnosed with MF. 3243 patients were male (60.0%). The median age was 69 ± 13 years (range 0–98 years old). Majority of the sample were NH-White (*n* = 4027, 74.5%) followed by NH-Black (*n* = 447, 8.3%), NH-Asian/Pacific islander (*n* = 408, 7.6%), NH-Native Alaskan/American Indian (*n* = 26, 0.5%), NH-others/unknown (*n* = 64, 1.4%) and Hispanic of all races(*n* = 431, 8%). Mean age among Black patients was 63.8 years compared to 68.6 years in non-Blacks (t = 7.233, *p* < 0.001). A summary of the demographics is provided in Table 1.

**Table 1:** Frequencies and multivariate Cox regression survival analysis for the SEER cohort

Variables		*n* (%)	Overall mortality		Cause specific mortality	
			HR (95% CI)*	*p*-value	HR (95% CI)*	*p*-value
Age (years)	< 35	81 (2%)	--	--	--	--
	35–65	1964 (36%)	2.5 (1.57-4.00)	**< 0.001**	2.56 (1.41–4.66)	**0.002**
	> 65	3358 (62%)	6.7 (4.23–10.73)	**< 0.001**	5.62 (3.10-10.19)	**< 0.001**
Sex	Female	2160 (40%)	--	--	--	--
	Male	3243 (60%)	1.4 (1.31–1.51)	**< 0.001**	1.40 (1.28–1.54)	**< 0.001**
Race	NH-White	4025 (75%)	--	--	--	--
	NH-Black	447 (8%)	1.19 (1.05–1.35)	**0.007**	1.07 (0.90–1.27)	0.435
	NH-Asian/Pacific Islander	408 (8%)	0.83 (0.73–0.95)	**0.008**	0.82 (0.69–0.98)	**0.028**
	NH-Alaskan/American Indian	26 (1%)	1.61 (0.98–2.63)	0.059	1.71 (0.94–3.10)	0.077
	Hispanics	431 (8%)	1.11 (0.98–1.26)	0.107	1.07 (0.91–1.26)	0.404
Year of Diagnosis	Pre 2011	2186 (41%)	--	--	--	--
	2011 or after	3217 (59%)	0.84 (0.79–0.91)	**< 0.001**	0.81 (0.74–0.89)	**< 0.001**
Income	<$70,000	2497 (46%)	--	--	--	--
	≥$70,000	2904 (54%)	0.92 (0.86–0.98)	**0.018**	0.96 (0.87–1.04)	0.309
Marital Status	Single	2397 (44%)	--	--	--	--
	Married	3006 (5403)	0.86 (0.81–0.93)	**< 0.001**	0.93 (0.85–1.02)	0.120

*Hazard ratio (95% confidence interval)

The age-adjusted incidence rate (95% CI) of MF was 0.32 (0.31–0.33) cases per 100,000 person-year. The annual percentage change significantly increased during the study duration from 2000 to 2021 (APC = 1.3%, 95% CI [0.1-2.5%], *p* < 0.05). At the time of the study, 3397 patients were censored (62.9%). The leading cause of death among the study population was attributed to MF (*n* = 2006, 37.1%), followed by cardiac diseases (*n* = 380, 7%), pulmonary diseases (*n* = 183, 3.4%) and GI diseases (*n* = 67, 1.2%).

**Fig. 1:**
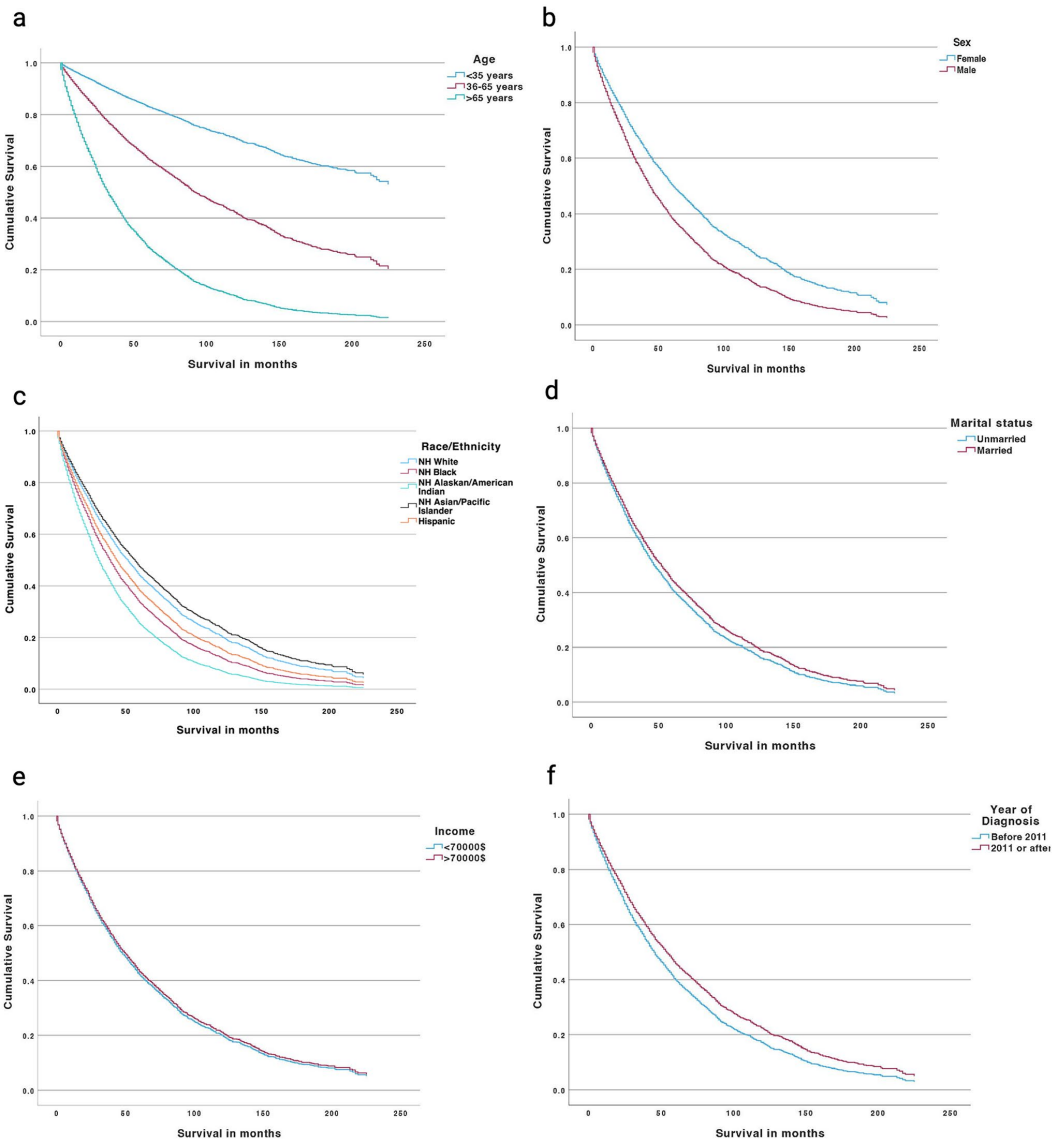
Cox regression overall survival estimates based off SEER variables (**a**) age, (**b**) sex, (**c**) ethnicity, (**d**) marital status, (**e**) income, and (**f**) year of diagnosis

Estimated overall survival was 69% and 42% at 2- and 5- years, respectively. The median overall survival was 46 months. Multivariable Cox regression analysis which included age, sex, ethnicity, marital status, income, and year of diagnosis (pre- and post- 2011) was performed on SEER data to estimate hazard ratio (HR) for cause-specific and overall mortality (Table 1). The overall survival models for the aforementioned regression are displayed in Fig. 1. Worse cause-specific survival was noted in older age, male sex, and those diagnosed before 2011. Asian/pacific islanders (API) showed improved cause specific survival. Marital status, black race and income were not significant predictors of cause-specific survival. However, they were associated with overall survival. Married patients and those with income higher than the median household income in the United States ($70,000) had higher median overall survival while black patients had worse overall survival.

### Single center cohort

Our single center cohort analysis included 84 complete cases. There were approximately equal males (51%) and females (49%), and median age at diagnosis was 66 years (range 29–88 years). Ethnic breakdown of demographic and clinical variables is displayed in Table 2. NH-White patients comprised the largest proportion of the individual races at 36.9%, followed by NH-Black (28.6%), Hispanic (26.2%), and NH-API (8.3%). Characteristics which differed significantly between races include sex (*p* = 0.037), presence of high molecular risk (HMR) mutations (*n* = 74; *p* = 0.009), and mean SVI (*p* < 0.001). Mean age at diagnosis varied amongst the different races, approaching significance (*p* = 0.059) with API patients being diagnosed earlier relative to others. Differences in the DIPSS score between races and mean hemoglobin at diagnosis also approached but did not reach significance (*p* = 0.062, *p* = 0.066 respectively).

**Table 2:** Ethnic breakdown of demographic and clinical variables from the single-center cohort

Variables		Non-Hispanic White (*n* = 31)	Non-Hispanic Black (*n* = 24)	Hispanic of all Races (*n* = 22)	Asian/Pacific Islanders (*n* = 7)	*p*-value*
Age, n (%)	< 65 years old (%)	12 (39)	11 (46)	9 (41)	6 (86)	0.156
	≥ 65 years old (%)	19 (61)	13 (54)	13 (59)	1 (14)	
	Mean age at dx	68.4	65.5	65.1	56	*0.059*
Sex, n (%)	Male	22 (71)	8 (33)	10 (45)	3 (43)	**0.037**
	Female	9 (29)	16 (67)	12 (55)	4 (57)	
Marital Status, n(%)	Single	19 (61)	13 (54)	15 (68)	4 (57)	0.81
	Married	12 (39)	11 (46)	7 (32)	3 (43)	
# of events (death)		13	9	7	0	
Mean survival (years)		8.7	7.7	9.4	NR^1^	0.2
Mean follow up (months)		55.3	37.7	44.5	53.3	0.390
HMR^2^ (*n* = 71), n(%)	None	22 (79)	9 (47)	8 (42)	5 (100)	**0.009**
	Present	6 (21)	10 (53)	11 (58)	0 (0)	
Mean hgb at diagnosis (g/dL)		9.79	8.65	10.4	9.87	*0.066*
DIPSS (*n* = 84)	Low risk	3	1	4	1	*0.062*
	Intermediate-1 risk	9	8	9	5	
	Intermediate-2 risk	19	13	6	1	
	High risk	0	2	3	0	
	Mean score	2.52	2.79	2.45	1.43	
Mean SVI score^3^		0.40	0.92	0.94	0.86	**< 0.001**

*p-value calculated from fisher’s exact test for categorical variables and ANOVA/Kruskal-Willis for continuous variables

^1^Not reached

^2^High molecular risk by MIPSS70v2 criteria including ASXL1, EZH2, SRSF2, IDH1/2, and U2AF1

^3^Social vulnerability index

There was a total of 29 deaths during the follow-up period and estimated overall survival (OS) at 2- and 5- years was 90.2% and 60.5% respectively. Median survival in the API cohort was not reached. Of ethnicities with calculated median OS, Hispanics had the longest survival at 9.4 years, followed by NH-White at 8.7 years, and lastly NH-Black at 7.7 years. Results for the single-center univariate regression are displayed in Table 3. Race did not show any significance on univariate cox regression, and nor did sex, marital status, or SVI. Age ≥ 66 years (HR 2.33, *p* = 0.028) and DIPSS (HR 1.66, *p* < 0.001) were both significantly associated with worsened survival, even after adjusting for race, sex, marital status, and SVI.

**Table 3:** Single center demographics and univariate survival analyses

Variables		*n* (%)	HR (95% CI)*	*p*-value
Age (using median cutoff)	≤ 65 years old	43 (51)	--	--
	> 65 years old	41 (49)	2.33 (1.10–4.95)	**0.028**
Sex, n(%)	Male	43 (51)	--	--
	Female	41 (49)	0.64 (0.30–1.36)	0.246
Race	NH-White	31 (37)	--	--
	NH-Black	24 (29)	1.46 (0.61–3.50)	0.401
	Hispanic	22 (26)	0.88 (0.35–2.21)	0.781
	Asian	7 (3)	NA^1^	NA^1^
Marital Status, n(%)	Single	51 (61)	--	--
	Married	33 (39)	1.08 (0.51–2.27)	0.848
DIPSS score		84 (100)	1.66 (1.24–2.23)	**< 0.001**
SVI score^2^		84 (100)	1.42 (0.43–4.65)	0.57

*Hazard ratio (95% confidence interval)

^1^Not reached due to 0 events

^2^Social vulnerability index

## Discussion

Our study shows that social and demographic factors including sex, race, marital status and income, are associated with survival in myelofibrosis. To our knowledge, this is the first study to compare national MF outcomes to that of an underserved minority-predominant cohort. The ethnic composition of residents in the Bronx is comprised of 54.8% Hispanic/Latino, 28.5% non-Hispanic Black, 8.9% non-Hispanic White and 4.6% non-Hispanic Asian [12]. More than a third of residents are born outside of the United States [13]. Additionally, the Bronx community faces unique social determinants of health challenges including high stress (62%), food insecurity (38%), unemployment (28%), physical limitations (23%), lack of transportation (21%), inadequate housing (19%), limited access to medical care (14%), and insufficient public services (11%) as per a 2017–2018 community survey of 608 respondents. Among the 62 counties of New York City, the Bronx has consistently ranked 62nd for health outcomes and carries a 26.4% poverty rate as of 2021. In 2020, Montefiore-Einstein Cancer Center identified a 22.5% rate of late-stage diagnosis of screen-detectable cancers which is 47% higher than the CDC’s 2015–2019 U.S. estimates [13].

Despite that, our single-center cohort showed better 2- and 5- year survival relative to the SEER cohort likely due to a combination of center-specific attributes and differences in study methodologies. Notably, a majority of patients in the MMC cohort were diagnosed after 2011, a factor associated with improved overall and cause-specific survival due to the approval of Ruxolitinib as a frontline treatment for MF [14]. Furthermore, unlike the SEER database, which aggregates data from various state registries and includes both community and academic centers [15], our cohort is based at a single academic comprehensive cancer center (CCC). Previous research has demonstrated that cancer outcomes at CCCs are generally superior to those at non-CCC institutions [16, 17]. Finally, variations in data collection and follow up processes between the two cohorts likely also explain some of the observed differences.

MF incidence throughout the study period was 0.32 cases per 100,000 person-year based on SEER data. The incidence of MF varies by several factors including age group, geographic location and timeframe. Multiple previous studies described similar incidence rates of MF over shorter time periods in the US [7, 18, 19], while European cohort studies reported a wider incidence range from 0.1 to 1.5 cases per 100,000 person-years [20]. These studies have also shown increasing trends of MF over time, similar to our findings in the US population [21].

Racial disparities in cancer outcomes are well described in many diseases including renal cell carcinoma, breast cancer, and head and neck cancer [22–24]. Interestingly, the SEER database analysis revealed worse overall but not cause-specific survival amongst NH-Black patients, while the NH-Asian cohort had improved overall and cause-specific survival relative to NH-White patients. These findings are present even after adjustment for other SDH factors including age, sex, income, marital status, and year of diagnosis (pre- and post- 2011). Regarding the single-center data, the MMC cohort is notably more diverse with only 37% of the cohort being NH-White in comparison to SEER’s 82%. Though ethnicity overall was not associated with OS in the MMC cohort, NH-Black patients similarly had the poorest median survival, while Hispanics had improved survival relative to NH-White patients. NH-Black patients, in addition to having the poorest outcomes, tended to carry a higher proportion of HMR mutations, have higher female predominance, present at a younger age with lower mean hemoglobin, and present with higher DIPSS score relative to other ethnicities. NH-Asian patients, on the other hand, had the best outcomes with no events reached during the follow-up period in our single-center cohort. They also presented at younger ages, but had lower mean DIPSS scores and carried the lowest proportion of HMR mutations in comparison to the other ethnicities. Furthermore, we found that NH-Black and Hispanic patients tended to live in areas with a higher level of vulnerability based on the CDC/ATSDR social vulnerability index, which accounts for various factors that adversely impact communities including poverty, unemployment, crowding, single-parent households, and lack of insurance [11]. These observed single-center disparities along with the SEER analysis suggest potential underlying biological and social factors which warrant further investigation and confirmation. In the case of NH-Black patients specifically, the lack of cause-specific survival association in the SEER analysis suggests that social factors may play a more prominent role in contributing to poorer outcomes, rather than disease-intrinsic factors. Contrary to our single-center analysis which found relatively higher proportion of HMR mutations in the NH-Black cohort, another recent multicenter study in the US found similar genetic profiles between black and non-black patients with MF. This study also noted worse post-transplant outcomes in the black cohort, possibly due to differences in donor source [8]. In comparison, the improved cause-specific and overall survival of NH-API patients in the SEER database, along with the observational single-center cohort findings, suggest that disease intrinsic factors may also play a role in their improved outcomes. Our results align with another study by Xu et al. which compared a cohort of Chinese MF patients to a western predominantly Caucasian cohort, and similarly found that the Chinese cohort presented at much younger ages, had lower DIPSS risk scores, and improved survival [25]. The study also noted other clinical differences in the Chinese cohort such as less hepatosplenomegaly and constitutional symptoms but more anemia, thrombocytopenia, and leukocytosis at diagnosis.

Concerning gender, the SEER data analysis found that being Male was associated with both worse overall and cause-specific survival. It is known that MF has a male preponderance of 3:2 as described in previous cohort studies [26]. This survival difference is less surprising based on previous studies which have similarly shown that females have better survival in myelofibrosis as well as other myeloproliferative neoplasms [10, 27]. From a biological point of view, females have lower JAK2V617F allele burden than males which may reflect sex-based mitotic recombination frequency [28]. Moreover, men exhibit markedly shorter telomere length compared to females irrespective of JAK2V617F status. This could indicate lower genomic stability in males compared to females that translates into slower progression [10, 29]. Regarding other demographic and socioeconomic factors, our SEER data analysis revealed that being unmarried and having lower income were associated with worse overall survival but not cause-specific survival. This is consistent with the broader literature documenting the impact of marital status on overall survival in other malignancies as well [30–33]. Studies suspect possible explanatory reasons include that married patients present earlier, are more likely to comply with treatment and have better psychological support and healthier eating habits [31]. Similarly, median income is a widely investigated environmental factor affecting survival in patients with cancer [34]. Our SEER analysis revealed that Black patients had significantly lower income than non-Black counterparts, providing another possible reason for the disparities in survival. And while our findings indicate that marital status and income are not significantly associated with cause-specific survival in myelofibrosis, it is crucial to acknowledge their substantial impact on overall survival. These factors likely influence broader health outcomes and access to care, which in turn affect disease management and patient longevity. Therefore, addressing these socio-demographic determinants is essential for improving comprehensive health outcomes and ensuring equitable care for all patients with myelofibrosis.

Our study’s findings must be interpreted within the context of its limitations. First, the reliance on recorded data could lead to information bias in variables prone to underreporting or misclassification, especially given recent evidence that MPNs are heavily underreported in US cancer registries [35]. Second, the heterogeneity in data sources, data collection methods, and follow-up procedures between SEER and the single-center cohort could lead to inconsistencies in reported outcomes and are important considerations when making direct comparisons. There is also difficulty of labeling disease-specific causes of death in MF, given that this condition increases the risk of death from common causes in the general population, such as vascular or cardiac ischemic complications, among others. Furthermore,it is not possible to rule out that the differences in MF incidence and overall survival demonstrated in the most recent period are not a consequence of changes in the WHO diagnostic criteria over time. Specifically, the inclusion of the prefibrotic form of MF in the latest period, which has a better prognosis than the established form of MF. Lastly, the small size of the single-center cohort limits the statistical power of our analysis and precludes the ability to conduct a robust multivariate analysis. Consequently, the single-center findings should be considered primarily observational, given that definitive conclusions cannot be drawn from this dataset alone.

In conclusion, our study highlights the complex interplay between demographic and socioeconomic factors in influencing survival outcomes in MF and contributes to the ongoing conversation about equity in healthcare outcomes. Future research should aim to validate these findings in larger, more diverse cohorts and explore the underlying mechanisms driving survival discrepancies that exist for non-disease related factors.

## Data Availability

Data for the SEER database is accessible upon submitting an application via the Data Request System (https://seerdataaccess.cancer.gov/seer-dataaccess) and installation of the SEER*STAT software (https://seer.cancer.gov/seerstat/software/). The link for the requisite version of the SEER database used (SEER 17 November 2022 submission) used is provided here under rate sessions: https://seer.cancer.gov/data-software/documentation/seerstat/nov2022/. The single-center data for this study are not openly available due to reasons of sensitivity and are available from the corresponding author upon reasonable request.

## Declarations

### Data sharing Statement

For original data, please contact the corresponding author Dr. Swati Goel at swgoel@montefiore.org.
